# Case Report: Preliminary Images From an Electromagnetic Portable Brain Scanner for Diagnosis and Monitoring of Acute Stroke

**DOI:** 10.3389/fneur.2021.765412

**Published:** 2021-10-29

**Authors:** David Cook, Helen Brown, Isuravi Widanapathirana, Darshan Shah, James Walsham, Adnan Trakic, Guohun Zhu, Ali Zamani, Lei Guo, Aida Brankovic, Ahmed Al-Saffar, Anthony Stancombe, Alina Bialkowski, Phong Nguyen, Konstanty Bialkowski, Stuart Crozier, Amin Abbosh

**Affiliations:** ^1^Intensive Care Unit, Princess Alexandra Hospital, Brisbane, QLD, Australia; ^2^Neurology, Princess Alexandra Hospital, Brisbane, QLD, Australia; ^3^School of Information Technology and Electrical Engineering, University of Queensland, Brisbane, QLD, Australia

**Keywords:** mobile brain scanner, electromagnetic neuroimaging, mobile stroke imaging, mobile neuroimaging, electromagnetic stroke imaging

## Abstract

**Introduction:** Electromagnetic imaging is an emerging technology which promises to provide a mobile, and rapid neuroimaging modality for pre-hospital and bedside evaluation of stroke patients based on the dielectric properties of the tissue. It is now possible due to technological advancements in materials, antennae design and manufacture, rapid portable computing power and network analyses and development of processing algorithms for image reconstruction. The purpose of this report is to introduce images from a novel, portable electromagnetic scanner being trialed for bedside and mobile imaging of ischaemic and haemorrhagic stroke.

**Methods:** A prospective convenience study enrolled patients (January 2020 to August 2020) with known stroke to have brain electromagnetic imaging, in addition to usual imaging and medical care. The images are obtained by processing signals from encircling transceiver antennae which emit and detect low energy signals in the microwave frequency spectrum between 0.5 and 2.0 GHz. The purpose of the study was to refine the imaging algorithms.

**Results:** Examples are presented of haemorrhagic and ischaemic stroke and comparison is made with CT, perfusion and MRI T2 FAIR sequence images.

**Conclusion:** Due to speed of imaging, size and mobility of the device and negligible environmental risks, development of electromagnetic scanning scanner provides a promising additional modality for mobile and bedside neuroimaging.

## Key Results

Electromagnetic imaging is an emerging technology which provides a mobile, and rapid neuroimaging modality for pre-hospital and bedside evaluation of patients based on the dielectric properties of the tissue.

The dielectric maps present anatomical structures, and machine learning algorithms provide localization and characterization of stroke type, which can be developed to guide future therapy and triage decisions.

An electromagnetic stroke scanner provides an additional modality for mobile and bedside neuroimaging.

## Summary Statement

This case report presents preliminary permittivity maps and images of stroke patients obtained using a rapid, portable low energy electromagnetic scanning device.

## Introduction

Electromagnetic imaging is an emerging technology which promises to provide a new, mobile, and rapid neuroimaging modality for pre-hospital and bedside evaluation of stroke patients. It is now possible due to technological advancements in materials, antennae design and manufacture, rapid portable computing power and network analyses and development of processing algorithms for image reconstruction (1–3). While research presented is addressing the challenges of improving acute stroke care, this electromagnetic imaging modality has potential for wider neuroimaging and other applications.

Mobile neuroimaging would create new therapeutic opportunities in the pre-hospital management, appropriate transport and ongoing monitoring of patients. Imaging to differentiate between ischaemic and haemorrhagic stroke is critical before instituting timely and effective treatments of acute stroke (4). Hyperacute stroke therapy such as thrombolysis or endovascular clot retrieval can minimize damage to salvageable ischaemic penumbra, but clinical outcomes are critically time dependent (5, 6), and thrombolysis can induce fatal bleeding if used in haemorrhagic stroke. Anticoagulant reversal, blood pressure control and anti-fibrinolytic treatments could limit intracranial hemorrhage volume, but for an ischaemic stroke patient, these could extend ischaemic injury. Mobile and portable imaging could transform the management of acute stroke in pre-hospital settings by allowing early treatment and triage to specialist stroke centers (7, 8). For every minute of treatment delay with thrombolysis, a median of 1.8 days of healthy life is lost (9). Early distinction between ischaemic and haemorrhagic stroke using mobile imaging could transform the management of stroke in the pre-hospital setting. Currently, mobile stroke units (MSU) are custom built ambulances equipped with CT scan and thrombolysis capability have been shown to lower disability at 3 months, compared to conventional ambulance based care (10, 11).

Electromagnetic imaging provides an opportunity for small rapid and portable neuroimaging to supplement the current imaging modalities. For the purpose of electromagnetic stroke imaging, the head contains a heterogeneous structure of tissues, fluids and interfaces with different *dielectric* properties which permit localization and characterization of internal structures and pathology (12). Electromagnetic imaging is based on characteristic and recognizable dielectric properties of tissues. These physical tissue properties are different to those properties that allow tissue characterization and anatomical mapping in X-ray, nuclear medical, MRI, ultrasound, impedance or electrical-potential based imaging or diagnosis. This is an example of translational research which poses technical challenges to develop the hardware and analysis, but with this comes clinical opportunities and an alternative window through dielectric physical properties to perform neuroimaging (13).

The dielectric properties of the tissues are the important physical properties which cause these effects, and these vary across the frequency band. For example, permittivity changes the velocity of signal propagation, whereas the tissue conductivity attenuates the signal. Typically tissues which have high water content will exhibit high permittivity, whereas conductivity increases for tissues with higher ionic salt content (14). In the context of stroke imaging, intracranial hemorrhages have a permittivity around 10–20% higher than the average permittivity of the brain. In contrast, in the early stages of ischemia, the tissue has about 10–20% lower permittivity than the average brain permittivity (12). Lastly, lesions with oedema have a higher permittivity than haemorrhagic lesions.

The aim of this report is to present preliminary images from a portable electromagnetic neuroimaging device in acute stroke.

## Materials and Methods

A prospective study of electromagnetic imaging was conducted on hospital in-patients with a confirmed diagnosis of either ischaemic or haemorrhagic stroke and with contemporaneous imaging, after informed written consent by patient or their surrogate decision maker. The non-interventional study was approved by the Health District Human Research Ethics Committee. The investigational imaging did not modify the patient treatment pathway. The purpose was to refine scanning algorithms for stroke neuroimaging.

Patients were included if over 18 years, had a radiological evidence of ischaemic or haemorrhagic stroke and were diagnosed in the previous 72 h. Patients were excluded if they were pregnant or lactating, had a history of seizures, or had metallic implants, surgical clips or devices, pacemakers, or surgical drains in the imaging field, or if the patient had a medical condition that would not allow for placement of the scanner or could not otherwise comply with scanning procedure.

The images were obtained by processing signals from encircling transceiver antennae which emit and detect low energy signals in the microwave frequency spectrum between 0.5 and 2.0 GHz. Information is acquired from the scattered, reflected and propagated signals arising from their complex interaction with the tissues. This information is captured with antennae taking turns transmitting and receiving with the antennae capturing its own transmitted signal and the signals of all others. Scattering occurs particularly at the interfaces between the tissues, and this is exhibited in the received magnitude and phase.

**Figure 1:**
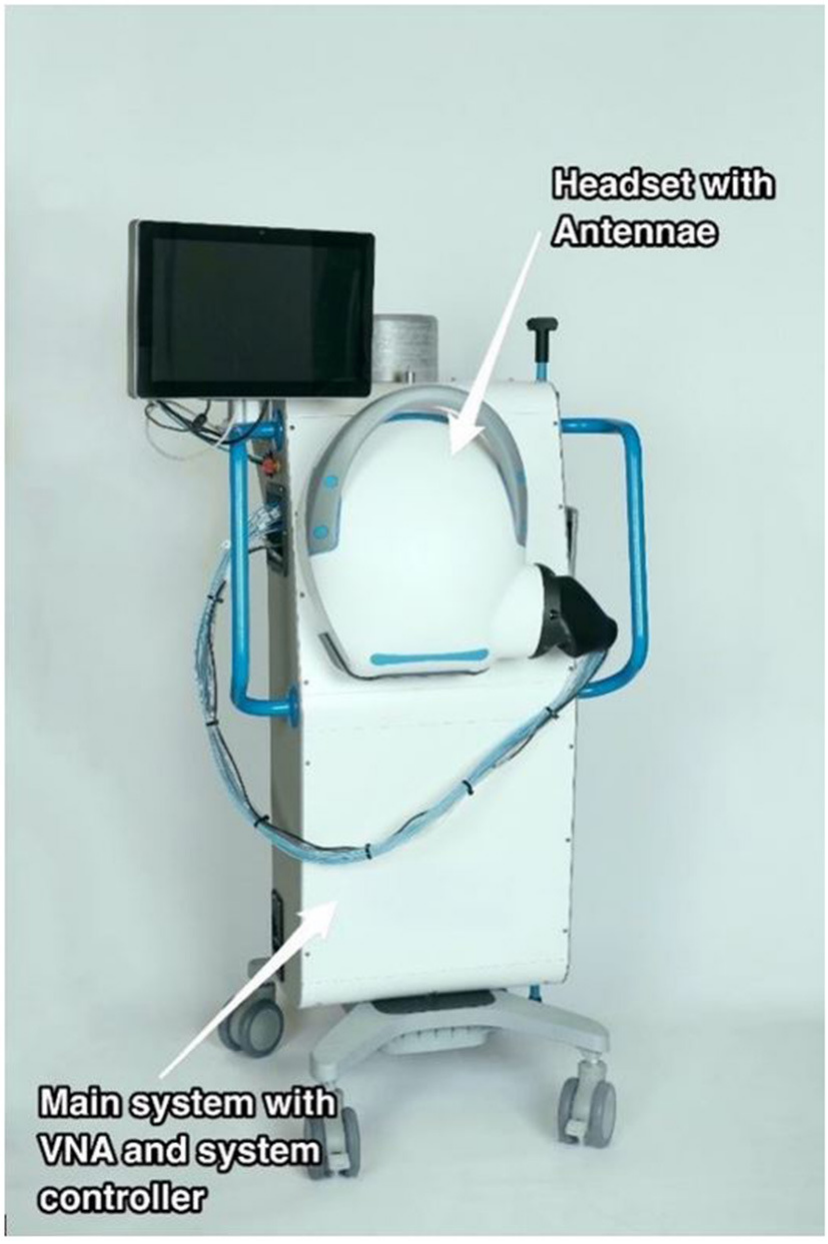
Clinical prototype electromagnetic head scanner.

The prototype mobile device (EMVision Medical Devices, Sydney, Australia, Figure 1) is similar in size to a portable ultrasound machine. The scanner has an antennae array in the helmet, with coaxial connections to the vector network analyser (VNA) and device controller with computer interface and screen. The antennae are shielded, preventing ingress of environmental electromagnetic radiation and preventing dispersion of internally generated signals. A coupling fluid filled membrane ensures the continuity between the head to be imaged, and the transceivers. The device is mains electricity powered but the transmitted microwave energy is very low at 10 mW. Data acquisition takes about 2 sec for all antennae to scan across the frequency spectrum in duplicate. There is a wealth of information embedded in the electromagnetic signals across the scanned range of frequencies, permitting analysis to exploit a diversity of approaches. The image reconstruction, *via* multiple algorithms takes seconds to a few minutes.

The scan is performed by the operator with the patient supine or with up to 30 degrees elevation of the head of the bed. The helmet is positioned on the head and the membrane of coupling fluid is filled. The scan is silent when performed and requires the patient to be still for 2 sec during scanning across the wide frequency band. When the scan is completed, the coupling fluid is pumped from the membrane, and the helmet is removed from the patient. The apparatus is cleaned, and the helmet returned to the transport position on the device.

The mobile electromagnetic scanner pictured in Figure 1 is a clinical prototype under evaluation at the Research Hospital with approval by the institutional Human Research Ethics Committee. It is not currently licensed for clinical use.

The differences in dielectric properties within the brain permit localization and characterization of internal structures, pathology and potentially function. These are physical properties of tissues that differ from the physical properties that form the basis for X-ray, magnetic resonance, sonographic or nuclear medical imaging. Although the anatomical structures will align, the appearance under electromagnetic scanning will differ.

**Figure 2:**
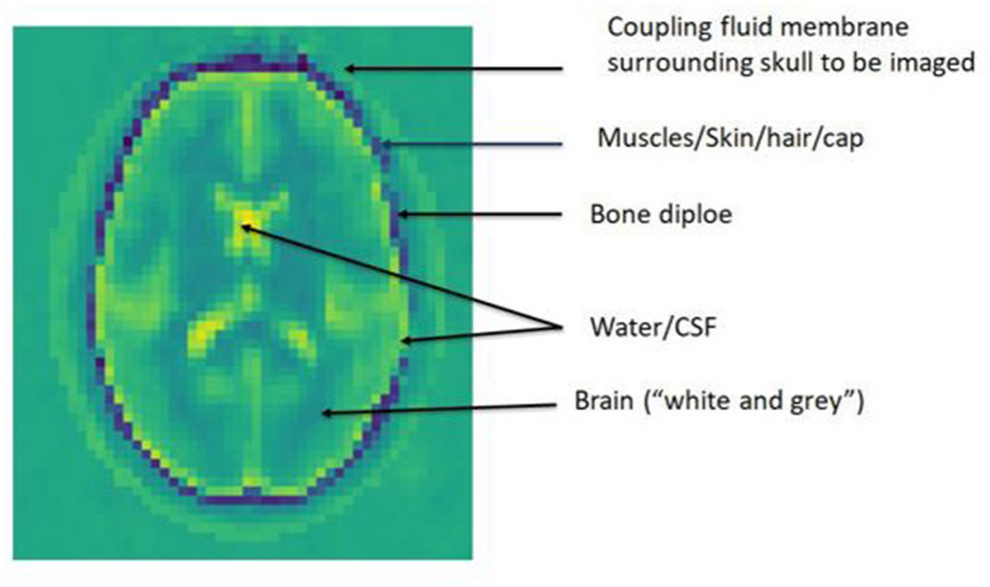
Permittivity map showing anatomical structures.

Figure 2 demonstrates an ~3 mm section with dielectric permittivity map displaying the anatomical structures imaged (15). There is no pathological lesion in this image. The variation in permittivity outlines the structures. The darkest regions are bone which has low permittivity. In this section, cerebrospinal fluid in the ventricles, fissures and over the brain surface is pale and has highest permittivity. Brain tissue (showing white and gray aspects), extracranial muscles and blood have intermediate values. The resolution is 2–4 mm which is less than CT or MRI.

**Figure 3:**
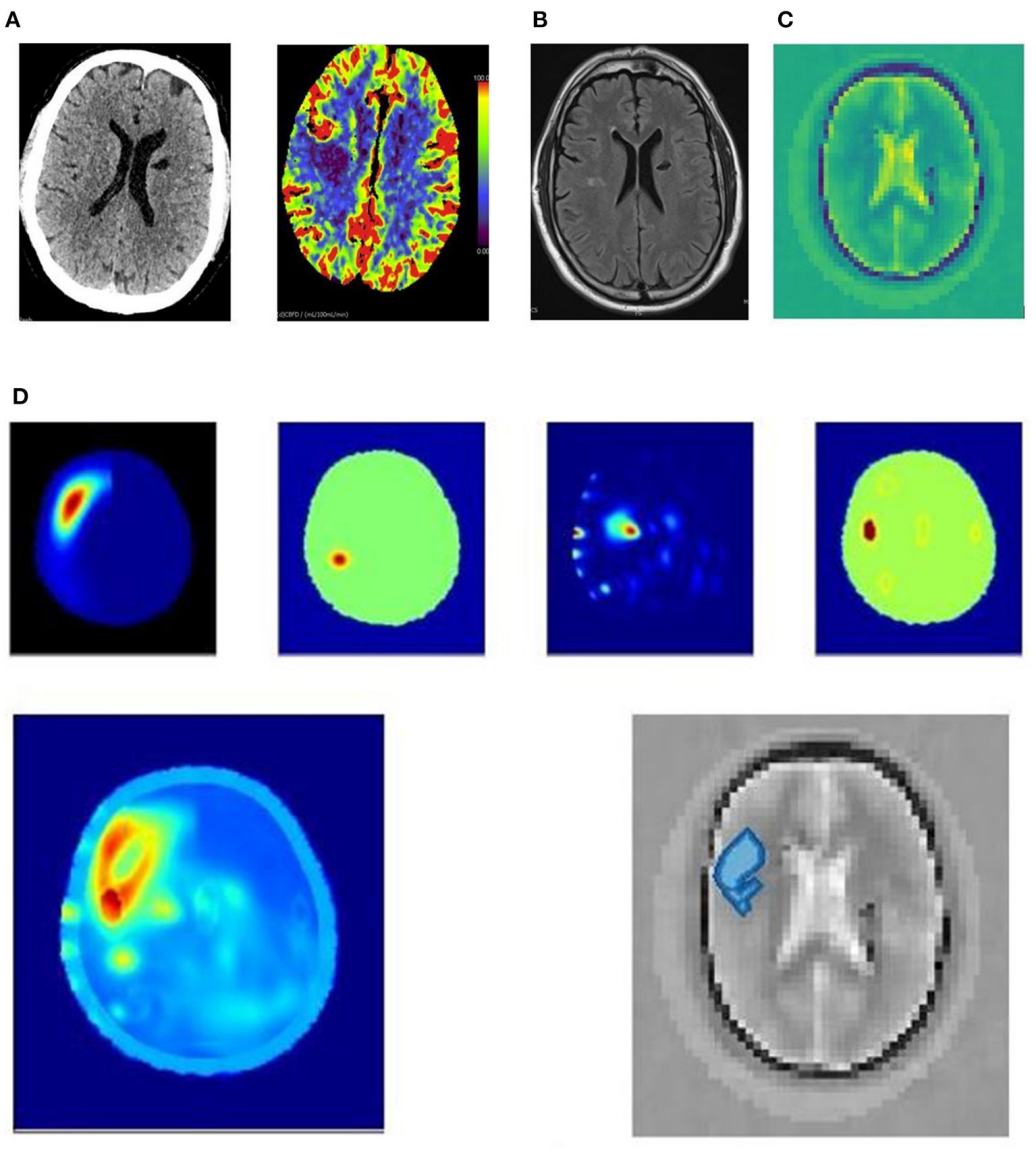
**(A–D)** 63yo patient with an ischemic stroke right middle cerebral artery distribution. **(A)** 63yo patient with an ischemic stroke right middle cerebral artery distribution. CT 3 hrs after onset of left hand weakness. The plain CT shows no acute abnormality; however, the cerebral blood flow perfusion scan (units ml/100 ml/minute cerebral blood flow for perfusion colormap) shows decreased blood flow in right middle lobe. **(B)** 63yo patient with an ischemic stroke right middle cerebral artery distribution. MRI T2 FLAIR sequence shows small area of diffusion restriction at 24 h after symptom onset, and after almost complete resolution of symptoms. **(C)** 63yo patient with an ischemic stroke right middle cerebral artery distribution. Dielectric permittivity map from an electromagnetic scan performed at 21 h after symptom onset. **(D)** 63yo patient with an ischemic stroke right middle cerebral artery distribution. Examples of processing algorithm using radar-based frequency domain and time domain signal analysis, signal asymmetry, tomography and direct mapping. The algorithm outputs are fused into a representative image for localization. An artificial intelligence algorithm classifies haemorrhagic stroke with a red color code, while ischaemic stroke is color-coded as blue, and this is overlaid on a greyscale permittivity map.

**Figure 4:**
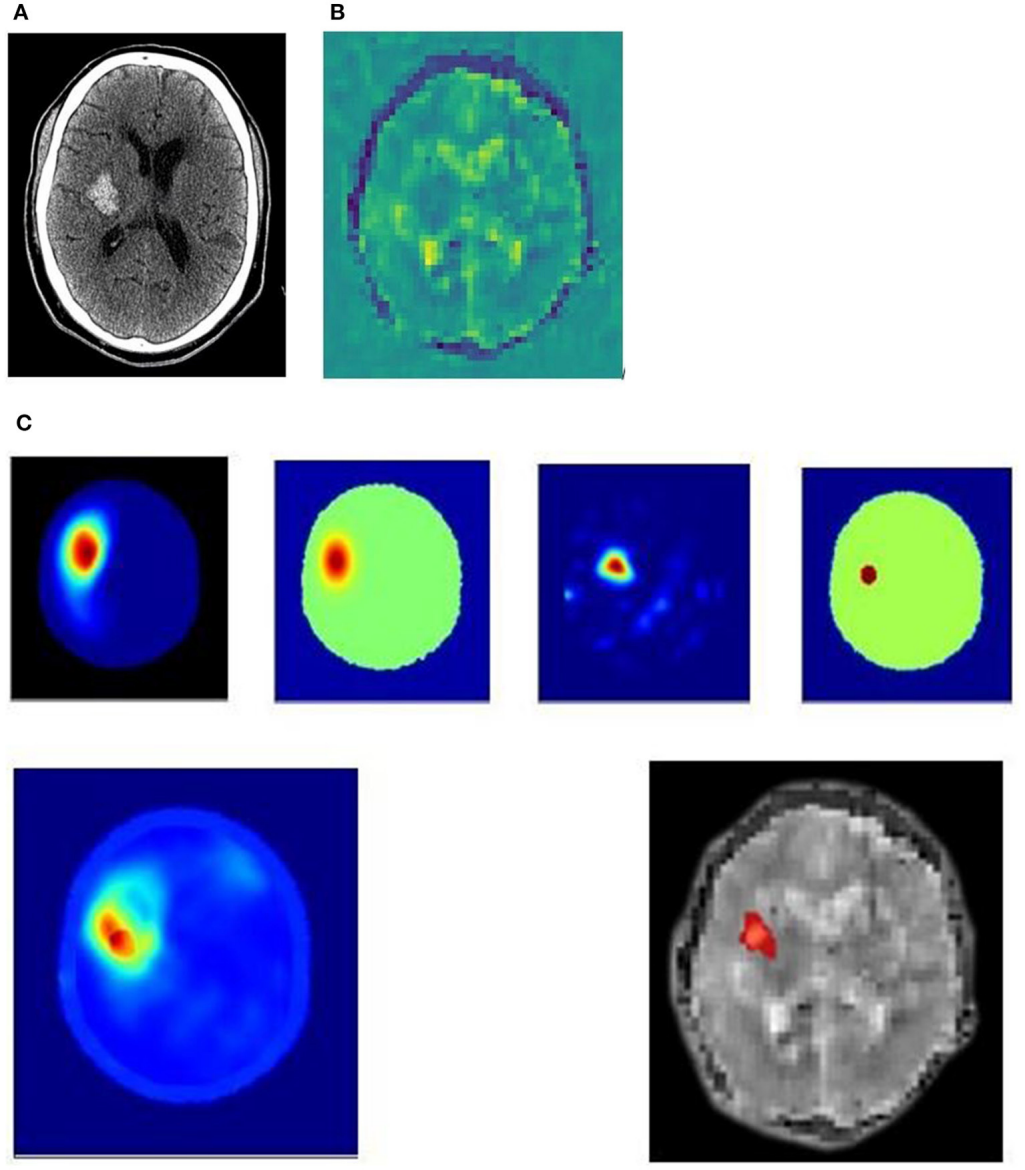
**(A–C)** 51yo patient with a hemorrhagic stroke, right basal ganglia. **(A)** 51yo patient with a hemorrhagic stroke, right basal ganglia. Non-contrast CT performed 2 hrs after onset of left paresis and facial droop, showing acute hemorrhage. **(B)** 51yo patient with a hemorrhagic stroke, right basal ganglia. Dielectric permittivity map from an electromagnetic scan performed 7 h after symptom onset. **(C)** 51yo patient with a hemorrhagic stroke, right basal ganglia. Examples of processing algorithm using radar-based frequency domain and time domain signal analysis, signal asymmetry, tomography and direct mapping. The algorithm outputs are fused into a representative image for localization. An artificial intelligence algorithm classifies haemorrhagic stroke with a red color code, while ischaemic stroke is color-coded as blue, and this is overlaid on a greyscale permittivity map.

The following Figures 3, 4 are examples of an ischaemic and an haemorrhagic stroke. In the panels, is the dielectric map of permittivity providing anatomical localization and five different electromagnetic scan algorithms. The electromagnetic scanning algorithms use diverse processing approaches including radar-based frequency domain and time domain signal analysis, signal asymmetry, tomography and direct mapping. The algorithm outputs can be fused into a representative image. In these image examples, an artificial intelligence algorithm classifies haemorrhagic stroke with a red color code, while ischaemic stroke is color-coded as blue. These images are compared to familiar diagnostic CT, CT perfusion and diagnostic MRI T2 FLAIR images.

Figure 3 is an example of a 63-year-old patient who presented with left hand weakness due to an acute right middle cerebral artery ischemic stroke. The plain and perfusion CT (Figure 3A) were performed 3 h after onset of symptoms and shows a narrow region of ischemia in the right posterior frontal lobe with little core infarction. An incidental, old left lacuna infarct is seen. Neither lysis nor clot retrieval was performed. The electromagnetic scan (Figures 3C,D) was performed 21 h after onset, and a subsequent MRI (Figure 3B) 24 h after onset shows a small area of infarction remains.

Figure 4 is an example of a 51-year-old patient who presented with left paresis and facial weakness. The CT (Figure 4A) was performed 2 h after onset of symptoms and shows a 33 × 21 × 23 mm acute haematoma in the right lentiform nucleus, with some surrounding cerebral oedema. An electromagnetic scan (Figures 4B,C) performed 7 h after symptom onset shows permittivity changes in the right basal ganglia, with a lower permittivity than the corresponding contralateral brain, suggestive of blood. Surrounding this is an area of higher permittivity, consistent with increased water content suggesting a surrounding rim of cerebral oedema.

## Discussion

The established stroke neuroimaging modalities (CT and MRI) provide excellent image quality but have limitations. They are large, heavy and require a stable physical platform, are expensive to purchase and maintain, and draw significant power. In addition to the clinical team, radiographers are necessary to safely perform the scans. Ionizing radiation, intravenous cannulation requirements and contrast media are drawbacks to CT. The magnetic fields produced by MRI pose environmental dangers and risks to patients with foreign bodies or devices. Where pre-hospital stroke diagnosis and management have been implemented, mobile stroke units (MSU) use down-sized conventional CT (16) and these are effective (10, 11). In hospital, mobile imaging using low resolution MRI for bedside use are promising and under evaluation (17).

An electromagnetic stroke scanner provides an additional adjunct modality for mobile neuroimaging. Devices are quick and portable, and tissue resolution is improving. Prehospital diagnosis, triage to specialist stroke units, and on-scene treatment are possible. It is the subject of ongoing work to establish the diagnostic capabilities of this emerging diagnostic modality, based on the dielectric properties of tissues.

The lower cost and portability of mobile electromagnetic scanning may overcome resource limitations where there are limited mobile CT based MSU assets (18). Electromagnetic scanning also promises applications in-hospital with bedside monitoring of patient progress, including therapeutic response, progress of oedema, reperfusion or haematoma, and complications such as haemorrhagic transformation or new ischemia.

## Conclusion

Due to speed of imaging, size and mobility of the device and negligible environmental risks, development of electromagnetic scanning scanner provides a promising additional modality for mobile and bedside neuroimaging.

## Data Availability Statement

The datasets of the cases presented in this article are not readily available due to ethical and privacy restrictions.

## Ethics Statement

The studies involving human participants were reviewed and approved by Metro South Human Research Ethics Committee, Princess Alexandra Hospital. The patients/participants provided their written informed consent to participate in this study.

## Author Contributions

DC, JW, KB, and AA: designed the trial. DC, HB, JW, IW, and DS: performed image acquisition. AT, ABr, GZ, AZ, ABi, AA-S, AS, KB, PN, LG, SC, and AA: analyzed the data. All authors contributed to writing or revising the manuscript.

## Funding

Research supported by Australian Cooperative Research Center Projects Grant CRCP-P60941, Advance Queensland Industry Research Fellowship AQIRF-063-2019RD2, and by industry research sponsorship (EMVision, Sydney). Conducted at the Princess Alexandra Hospital under institutional research ethics approval as a non-interventional observational study: HREC/2019/QMS/48520 An Early Feasibility Study to Obtain Imaging Data from Participants with a Diagnosed Stroke to Refine the Algorithm for the EMVision Brain Scanner.

## Conflict of Interest

AA, KB, ABi, SC, LG, PN, AZ, and GZ are authors of relevant patents. SC, KB, DC, and JW have non-executive or advisory positions with EMVision. SC, DC, DS, and JW are shareholders in EMVision. AA-S, LG, PN, AS, IW, AZ, and GZ are part funded by EMVision. The remaining authors declare that the research was conducted in the absence of any commercial or financial relationships that could be construed as a potential conflict of interest. The research was conducted to refine and develop the imaging technology. The authors declare that this study received funding from EMVision, Sydney, with involvement in the design, manufacture and supply of the device, the study design and analysis of the data.

## Publisher's Note

All claims expressed in this article are solely those of the authors and do not necessarily represent those of their affiliated organizations, or those of the publisher, the editors and the reviewers. Any product that may be evaluated in this article, or claim that may be made by its manufacturer, is not guaranteed or endorsed by the publisher.

## Abbreviations

MSU: Mobile Stroke Unit
VNA: Vector network analyser.
